# Protective effect of the ultra-filtration extract from Xin Mai Jia on human aortic smooth muscle cell injury induced by hydrogen peroxide

**DOI:** 10.3892/etm.2013.1365

**Published:** 2013-10-25

**Authors:** JIA WAN, YALING YIN, RUILI SUN, GUOPIN PAN, PENG LI, YANLONG JIA, GUANGRUI WAN, ZHANG-SUO LIU

**Affiliations:** 1 Department of Nephrology, The First Affiliated Hospital, Zhengzhou University, Zhengzhou, Henan 450052, P.R. China; 2 School of Basic Medical Sciences, Xinxiang Medical University, Xinxiang, Henan 453003, P.R. China; 3 Department of Inspection, Xinxiang Medical University, Xinxiang, Henan 453003, P.R. China; 4 College of Pharmacy, Xinxiang Medical University, Xinxiang, Henan 453003, P.R. China; 5 Modern Technology Education Center, Xinxiang Medical University, Xinxiang, Henan 453003, P.R. China

**Keywords:** Xin Mai Jia, human aortic smooth muscle cell, proliferation, migration, inflammation

## Abstract

The aim of the present study was to explore whether an ultra-filtration extract from Xin Mai Jia (XMJ), a Chinese medicinal formulation, has a protective effect on human aortic smooth muscle cell (HASMC) injury models induced by hydrogen peroxide (H_2_O_2_), and to consider the mechanism and efficacy of the therapeutic action of XMJ on atherosclerosis. HASMCs were injured by H_2_O_2_ and then exposed to various concentrations of XMJ. The morphological changes, growth, proliferation, migration and cytokine release of HASMCs were detected using 2,3-bis-(2-methoxy-4-nitro-5-sulfophenyl)-2H-tetrazolium-5-carboxanilide (XTT), an enzyme-linked immunosorbent assay and a scratch adhesion test. H_2_O_2_ significantly promoted the proliferation of HASMCs. The ultra-filtration extract from XMJ was observed to significantly attenuate the morphological changes of injured HASMCs, reduce the expression levels of intercellular adhesion molecule-1 (ICAM-1), vascular cell adhesion molecule-1 (VCAM-1), interleukin (IL)-1, IL-6 and nuclear factor (NF)-κB, and increase the expression levels of matrix metalloproteinase (MMP)-2 and tissue inhibitor of metalloproteinase (TIMP). XMJ has clear anti-inflammatory and antioxidant effects, and significantly inhibits the proliferation and migration of HASMCs.

## Introduction

Arteriosclerosis (AS) is a chronic inflammatory disease. Reactive oxygen species (ROS) are produced during exposure to oxidative stress, such as hydrogen peroxide (H_2_O_2_), and bind to the nuclear receptor of vascular endothelial cells and smooth muscle cells (SMCs) as ligands. ROS directly regulate the gene expression of various types of signaling molecules, such as interleukin (IL), intercellular adhesion molecule (ICAM) and vascular cell adhesion molecule (VCAM), to enhance the adhesion and migration of monocytes to the tunica intima, which is vital in the early stage of AS (1–3). Various types of cells and inflammatory mediators participate in the occurrence of AS (4–7), particularly the vascular smooth muscle cells (VSMCs) of the tunica media. The VSMCs of the tunica media form SMC-derived foam cells after proliferation, phenotypic transition and migration to the intima in the presence of a stimulating factor. The VSMCs of the tunica media are significant in the early, middle and advanced stages of AS, as well as in the pathogenesis of vascular stenosis diseases. VSMCs are categorized as important pathological features of AS (8–11).

Xin Mai Jia (XMJ) is a Chinese medicinal formulation that is available in capsule form. XMJ contains 10–35% functional red kojic rice powder, 1–10% kudzu flavonoid powder, 1–8% soybean isoflavone powder, 1–8% bamboo leaf flavone powder, 1–8% resveratrol powder, 1–6% hawthorn powder, 1–6% gastrodia powder, 1–30% *Auricularia auricula* powder, 0.1–0.2% hippocampus powder, 0.008–0.04% astaxanthin powder, 0.1–0.3% menthol powder and 20–50% resistant starch.

This health food contains several natural antioxidants, which enhance immunity and decrease the effects of ageing (12–15). A previous study has shown that XMJ is able to alleviate cardiovascular and cerebrovascular diseases, reduce blood lipids, normalize blood pressure, increase energy levels and improve sleep after several months of intake (16). Although the effect of XMJ is satisfactory, the exact mechanism of its antiarteriosclerotic action has not been confirmed.

## Materials and methods

### Drugs and chemicals

The crude components of XMJ were purchased from Beijing Tong Ren Tang Chinese Medicine Co. Ltd. (Beijing, China). Lovastatin, H_2_O_2_, 2,3-bis-(2-methoxy-4-nitro-5-sulfophenyl)-2H-tetrazolium-5-carboxanilide (XTT) and phenazine methosulfate (PMS) were purchased from Sigma Chemical Co. (St. Louis, Mo, USA). Zhibituo was obtained from Chengdu Diao Pharmaceutical Group Co., Ltd. (Chengdu, China). The RPMI-1640 culture medium and fetal calf serum were acquired from Gibco (Carlsbad, CA, USA). Wright-Giemsa (R-G) stain was purchased from Changsha Li Xin Biotechnology Co. (Changsha, China). IL-1, IL-6, ICAM-1, VCAM-1, matrix metalloproteinase-2 (MMP-2), tissue inhibitor of metalloproteinase (TIMP-2) and nuclear factor (NF)-κB were purchased from R&D Systems (Minneapolis, MN, USA). All other reagents were analytically pure and purchased in China.

### Cell experiment protocol

Human aortic smooth muscle cells (HASMCs) were obtained from HASMC cell lines purchased from American Type Culture Collection (Manassas, VA, USA). The cells were routinely maintained in phenol red containing Dulbecco’s modified Eagle’s medium supplemented with 15% fetal calf serum, 100 U/ml penicillin and 0.1 mg/ml phytomycin in a 37°C incubator containing 5% CO_2_. The third generation of HASMCs was used in the study. The cells were randomly divided into eight groups and incubated with the corresponding drugs for 24 h. The cells in the first group were incubated with Kreb’s solution and were assigned to the blank control group (n=6). The cells in the second group were incubated with 500 mg/l XMJ and were assigned to the XMJ control group (n=6). The cells in the third group were incubated with 200 μmol/l H_2_O_2_ and were assigned to the model group (n=6). The cells in the fourth group were incubated with 1 μmol/l lovastatin and 200 μmol/l H_2_O_2_ and were assigned to the lovastatin group (n=6). The cells in the fifth group were incubated with 50 μmol/l zhibituo and 200 μmol/l H_2_O_2_ and were assigned to the zhibituo group (n=6). The cells in the sixth group were incubated with 25 μmol/l XMJ and 200 μmol/l H_2_O_2_ and were assigned to the low-dose XMJ group (n=6). The cells in the seventh group were incubated with 50 μmol/l XMJ and 200 μmol/l H_2_O_2_ and were assigned to the middle-dose XMJ group (n=6). The cells in the eighth group were incubated with 100 μmol/l XMJ and 200 μmol/l H_2_O_2_ and were assigned to the high-dose XMJ group (n=6). The cultured cells were collected after each treatment for subsequent tests.

### Preparation of the ultra-filtration membrane extracts for XMJ

Approximately 1,000 g of the crude components of XMJ were placed in a container containing 6,000 ml water and heated for 1 h in a microwave oven at 1,000 W. The decoction of XMJ was obtained after filtering the extract through four gauzes. Thereafter, 6,000 ml water was added to the container and the above procedure was repeated. After mixing the former decoction of XMJ with the latter one, the mixture was filtered using sterile absorbent cotton. XMJ was refined by ultra-filtration technology with water decoction at a pressure of 0.5 kPa/m^3^, a temperature of 25°C, and a flow rate of 100 l/h/m^2^. Approximately 5,000 ml of filtrate was then condensed to 1,000 ml, which was equivalent to 1 g of XMJ/ml of liquid medicine. Finally, the refined liquid was labeled and stored in a refrigerator at 4°C.

### HASMC proliferation

Seed cells were placed into 96-well plates (100 μl/well) and cultured in RPMI-1640. Thereafter, the null medium was discarded and replaced with 100 μl fresh medium and 25 μl XTT and PMS mixture. After cell culture for 4 h, the optical density (OD) was measured at 450 and 630 nm wavelengths using an enzyme-linked immunosorbent assay (ELISA) plate reader (BioTek Instruments, Inc., Winooski, VT, USA). Each group contained six wells. The result was obtained from the average value of these six wells.

### Wright-Giemsa staining

The cells were plated in a 24-well plate at 5×10^4^ cells/ml, treated with XMJ (25, 50 and 100 μmol/l), lovastatin and zhibituo, respectively, for 24 h. The sterile slides were placed in a 24-well plate in advance for the adherence of cells. The slides were then taken out and washed twice with phosphate-buffered saline (PBS). After air-drying at room temperature, Wright-Giemsa dye was added to rapidly cover the slide. The same amount of PBS was added a few minutes later. The Wright-Giemsa dye and the PBS were mixed using a rubber pipette bulb. The dye was then rinsed with water, dried and sealed, and the cells were examined under a microscope (Olympus, Fukushima, Japan).

### Detection of biochemical indicators in HASMC supernatant fluid

Seed cells were placed into 6-well plates in 2 ml concentrations/well. To detect the biochemical indicator of the cell supernatant fluid, the culture fluid was collected to measure the superoxide dismutase (SOD) and malondialdehyde (MDA) activity, following the manufacturer’s instructions. The reagents and kit used to measure SOD and MDA were purchased from Nanjing Jiancheng Biological Engineering Institute (Nanjing, China).

### ELISA

Seed cells were placed into 6-well plates (2 ml/well). The supernatant fluid of the various wells in the plates was collected by an ELISA reagent kit, according to the manufacturer’s instructions (R&D Systems Inc., Minneapolis, MN, USA). The OD value of each well was measured at a 450 nm wavelength. The OD value was assigned as the abscissa, while the standard liquid concentration of the reagent was assigned as the ordinate. The relevant curve was drawn and the curve equation was calculated. The OD values of the samples were substituted into the equation of the standard curve, and the IL-1, IL-6, ICAM-1, VCAM-1, MMP-2, TIMP-2 and NF-κB values were calculated.

### HASMC transfer ability

Seed cells were placed into 24-well plates (1 ml/well). After 24 h of cell culture, a line of the same width was drawn in each well with a 200 μl pipette tip, and the plates were washed three times with sterile PBS. The cells were cultured in RPMI-1640 medium without serum, and exposed to XMF (25, 50 and 100 μmol/l), lovastatin and zhibituo, respectively, for 24 h. The entire process was recorded. The scratch width was detected using scratch image software 3.22 (Olympus). Six scratch belt widths were collected in each well, and the average value of the widths was calculated and compared.

### Statistical analysis

All data are shown as the mean ± standard error. Single factor variance and Student-Newman-Keuls multiple comparison analyses were used to compare data from different groups. Comparisons were performed using statistical software SPSS 13.0 (SPSS, Inc., Chicago, IL, USA. P<0.05 was considered to indicate a statistically significant result.

## Results

### Different concentrations of XMJ and H_2_O_2_ stimulate morphological changes in HASMCs

**Figure 1:**
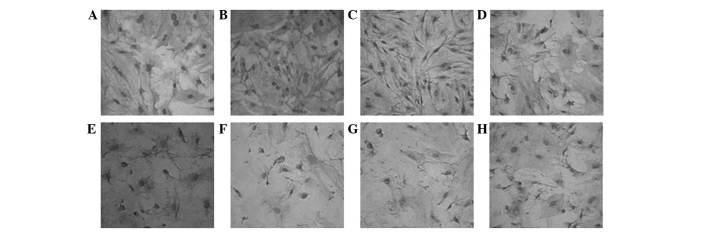
Different concentrations of XMJ and H_2_O_2_ stimulate HASMC morphological changes (magnification, ×200; R-G staining). (A) Blank control group; (B) XMJ control group; (C) model group; (D) lovastatin group; (E) zhibituo group; (F) low-dose XMJ group; (G) middle-dose XMJ group; (H) high-dose XMJ group. XMJ, Xin Mai Jia; HASMC, human aortic smooth muscle cell; R-G, Wright-Giemsa dye.

The HASMCs in the control group were arranged closely with abundant cytoplasms and intact cell membranes. The HASMCs demonstrated typical ‘peak-valley’-like growth. The number of cells in the normal group was lower than that in the model group. The normal group had loose cell connections and exhibited cytoplasm shrinkage, whereas the model group lost its typical growing appearance but had a fusiform cell presentation. The drugs inhibited cell proliferation to varying degrees. Certain damaged cells were restored with plumper cytoplasms and clearer profiles. However, these effects were much clearer in the high-dose XMJ group. Certain cells in the high-dose XMJ group reverted to a near-normal state. However, no significant effects were observed in the zhibituo and lovastatin groups. The cell shrinkage in the lovastatin group was particularly evident (Fig. 1).

### Inhibitory effects of XMJ on H_2_O_2_-induced HASMC proliferation

**Figure 2:**
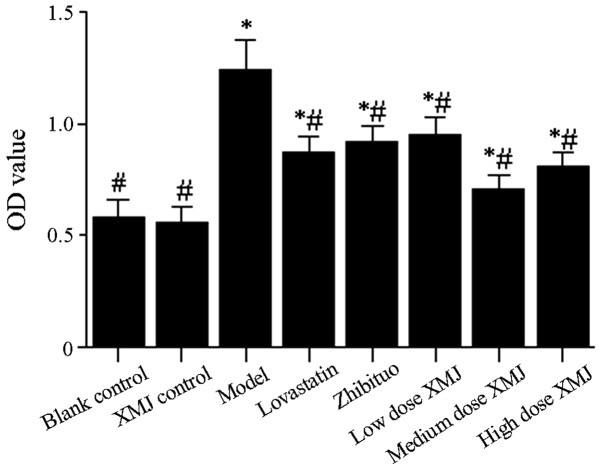
Inhibition of H_2_O_2_-induced HASMC proliferation by different concentrations of XMJ. Means ± SE, n=6. ^*^P<0.05 vs. blank control group; ^#^P<0.05 vs. model group. XMJ, Xin Mai Jia; HASMC, human aortic smooth muscle cell; OD, optical density.

Cells that proliferate after HASMC treatment are damaged and gradually migrated to the vascular intima from the tunica media vasorum, thus inducing plaque formation (17). Excessive proliferation of HASMCs is also an important factor in high blood pressure (18). The results showed that HASMCs in the model group were significantly more proliferative after 24 h of interaction than the cells in the normal group (Fig. 2, P<0.05). The results showed that HASMCs in the model group were significantly more proliferative after 24 h of interaction than the cells in the normal group, proliferative HASMCs in the middle-dose XMJ group, low-dose XMJ group and high-dose XMJ group were significantly inhibited after 24 h of interaction in a dose-dependent manner.

### XMJ anti-inflammatory effects

Inflammation induces atherosclerotic plaque formation and occurs during the development of AS. Therefore, we examined the inflammatory factors MMP-2 and TIMP-2 in the model group. The results showed that the levels of both factors were significantly decreased in the model group compared with those in the normal group (P<0.05); however, the levels of both factors increased significantly after treatment with different concentrations of XMJ compared with those in the model group (P<0.05). The most significant effect was observed in the middle-dose XMJ group. The levels of both factors also increased in the zhibituo and lovastatin groups; however, the effect was not significant compared with those in the XMJ groups (Table I).

### NF-κB promotes the release of various inflammatory factors

**Figure 3:**
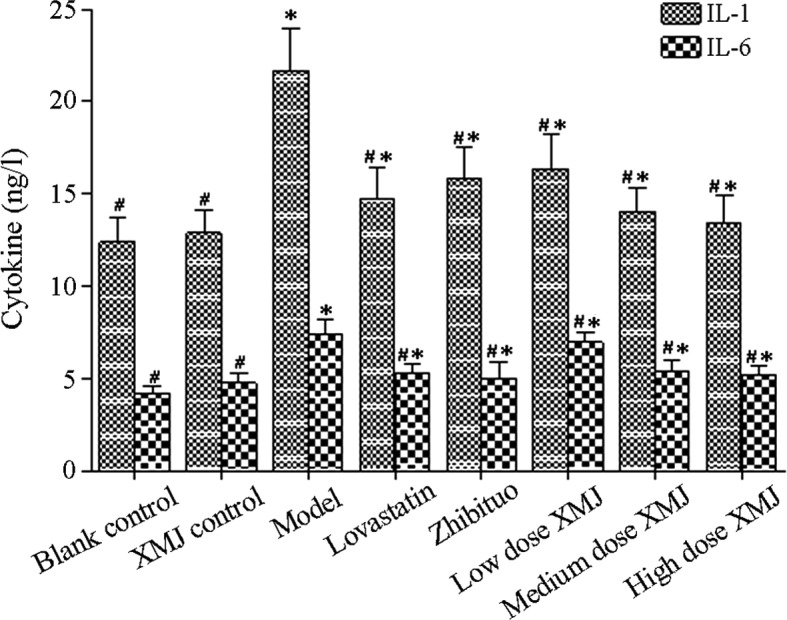
Changes in IL-1 and IL-6 levels in HASMCs induced by H_2_O_2_ after treatment with different concentrations of XMJ. Means ± SE, n=6. ^*^P<0.05 vs. blank control group; ^#^P<0.05 vs. model group. IL, interleukin; XMJ, Xin Mai Jia; HASMC, human aortic smooth muscle cell.

The activation mechanism of the NF-κB pathway and its effect on related inflammatory factor content (including IL-1 and IL-6 levels; Fig. 3) were investigated. The results showed that the NF-κB content in the model group was significantly increased compared with that in the normal group (P<0.05), thus indicating that the NF-κB pathway was significantly inhibited. However, the NF-κB content decreased significantly after treatment with different concentrations of XMJ compared with those in the model group (P<0.05). This result indicates that inhibition of NF-κB pathway activation by XMJ reduced the inflammatory response. The NF-κB content decreased significantly in the zhibituo group compared with that in the model group (P<0.05). No significant difference was observed between the zhibituo and XMJ groups. The NF-κB content also decreased in the lovastatin group compared with that in the model group (P<0.05). However, the effect of the lovastatin group was greater than that of the XMJ and zhibituo groups (Table I).

### XMJ antioxidant effects

**Figure 4:**
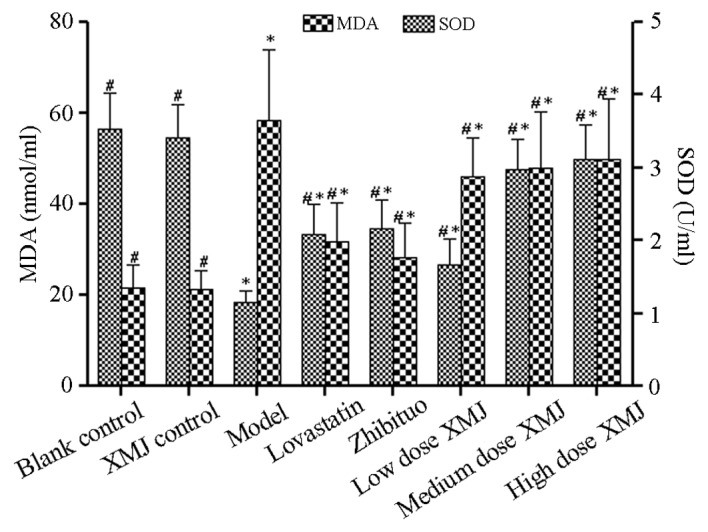
Antioxidant effects of XMJ. Means ± SE, n=6. ^*^P<0.05 vs. blank control group; ^#^P<0.05 vs. model group. XMJ, Xin Mai Jia; MDA, malondialdehyde; SOD, superoxide dismutase.

Lipid peroxidation induces plaque formation during AS development (19). Plaque formation may be controlled if lipid peroxidation is inhibited effectively. SOD is a well-known and important antioxidant enzyme. Therefore, we first detected the SOD activity (Fig. 4). The SOD activity increased significantly in a concentration-dependent manner after treatment with various concentrations of XMJ. The SOD activity was significantly different in the high-dose XMJ group from that in the model group (P<0.05). This result showed that XMJ may effectively promote the synthesis of antioxidant molecules such as SOD by HASMCs, thus effectively inhibiting the occurrence of oxidation. The MDA content, which is an important indicator of oxidative stress, was also measured. The MDA content in the model group increased significantly compared with that in the normal group, but decreased significantly after treatment with various concentrations of XMJ (P<0.05). The MDA content in the zhibituo and XMJ groups was significantly decreased compared with that in the model group (P<0.05), thus indicating that XMJ exerts antioxidant effects by inhibiting MDA synthesis.

### Anti-adhesion effects of XMJ inhibit HASMC migration

**Figure 5:**
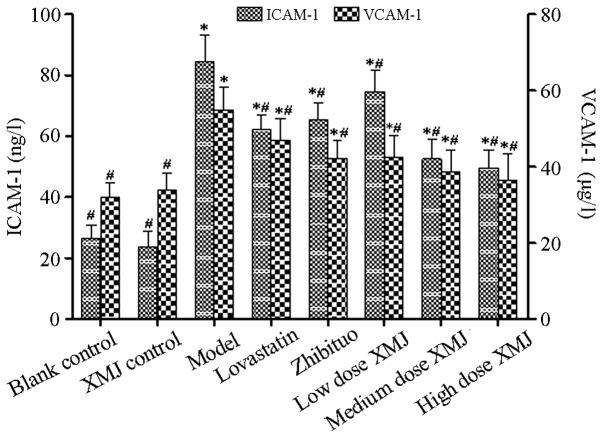
Changes in ICAM-1 and VCAM-1 levels in HASMCs induced by H_2_O_2_. after treatment with different concentrations of XMJ. Means ± SE, n=6. ^*^P<0.05 vs. blank control group; ^#^P<0.05 vs. model group. ICAM-1, intercellular adhesion molecule-1; VCAM-1, vascular cell adhesion molecule-1; XMJ, Xin Mai Jia; HASMC, human aortic smooth muscle cell.

**Figure 6:**
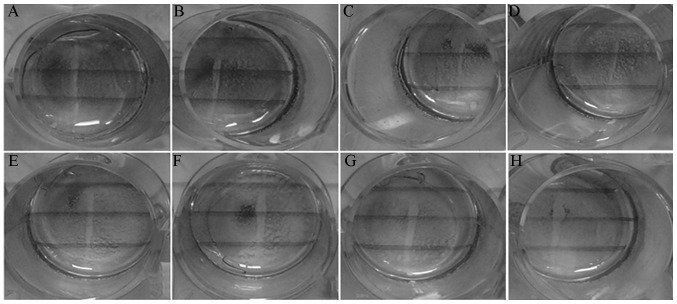
XMJ anti-adhesion to inhibit HASMC migration. (A) Blank control group; (B) XMJ control group; (C) model group; (D) lovastatin group; (E) zhibituo group; (F) H_2_O_2_ culture medium; (G) middle-dose XMJ group; (H) high-dose XMJ group. XMJ, Xin Mai Jia; HASMC, human aortic smooth muscle cell.

**Figure 7:**
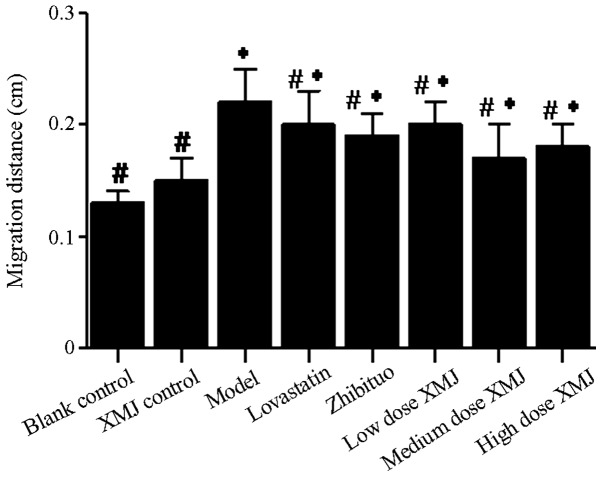
Migration distance of XMJ. Means ± SE, n=6. ^*^P<0.05 vs. blank control group; ^#^P<0.05 vs. model group. XMJ, Xin Mai Jia.

The proliferation and adhesion capacity of HASMCs from the tunica media to the tunica intima is enhanced in injured HASMCs (20). Therefore, the expression of adhesion factors ICAM-1 and VCAM-1 was detected (Fig. 5). The results showed that the levels of the two adhesion factors were significantly increased in the model group compared with those in the normal group (P<0.05), but decreased significantly in a concentration-dependent manner after treatment with different concentrations of XMJ compared with those in the model group (P<0.05). The ICAM-1 content in the zhibituo and lovastatin groups decreased significantly (P<0.05) as did the VCAM-1 content. These results showed that XMJ reduced the adhesion and migration of HASMCs from the tunica media to the tunica intima. The scratch experiment showed that the scratch width narrowed significantly in the model group, and that the cell migration ability became stronger (Fig. 6). The scratch width widened significantly after treatment with different concentrations of XMJ. Cells were shed from the cell wall due to the reduction in cell adhesion. No significant difference was observed between the scratch distances in the zhibituo and lovastatin groups compared with those in the model group (Fig. 7).

## Discussion

Contractile SMCs are present in the tunica media, the cytoplasms contain numerous myofilaments, but fewer organelles (21,22). The main function of SMCs is to regulate vascular tone. Inflammation and trauma reduce the specificity of telescopic protein expression, inducing a proliferative state in SMCs, increasing their migration and proliferation and promoting damage repair processes (23,24). The overexpression of the repair process in damaged blood vessels induces the development of vascular diseases such as AS, vascular stenosis and hypertension (25–27). During the development of AS lesions, VSMCs confer proliferative effects with migrative functions and promote the endometrial repair of vascular intima damage, thus resulting in restenosis and atherosclerotic plaque formation and growth (28–30). Therefore, the prevention of VSMC proliferation and aggregation may contribute to the prevention of restenosis and atherosclerotic plaque formation.

The results of the present study indicated that XMJ inhibited HASMC proliferation by inhibiting IL-1 and IL-6 expression. A reduction in the number of SMCs effectively decreases the tension of blood vessels and prevents vascular stenosis (31). AS is a chronic inflammatory disease. XMJ promoted TIMP-2 and MMP-2 expression by activating the NF-κB pathway. This may reduce the H_2_O_2_-induced HASMC injury and inhibit further cell damage by reducing the inflammatory response. This is likely to delay the development of disease and promote healing. SOD is important as an antioxidant. Increased SOD synthesis helps to reduce oxidative stress in the AS process (32). VCAM-1 and ICAM-1 are important in the migration of SMCs from the tunica media to the tunica intima (33). XMJ reduced the adhesion of HASMCs by inhibiting HASMC synthesis, thus effectively inhibiting HASMC migration. The results showed that XMJ promotes cell repair by inhibiting the proliferation, inflammatory response, adhesion and antioxidant mechanisms of HASMC and is likely to reduce plaque formation in AS. XMJ may play a protective role in AS. Further studies are required to determine whether the inhibition of intercellular adhesion expression causes plaque shedding and cardiovascular and cerebrovascular diseases.

## Figures and Tables

**Table I:** XMJ anti-inflammatory effects.

Groups	TIMP-2 (pg/l)	MMP-2 (μg/l)	NF-κB (ng/l)
Blank control	1987.36±125.39^a^	0.682±0.09^a^	44.98±7.89^a^
XMJ control	1875.41±115.47^a^	0.675±0.08^a^	46.66±8.47^a^
Model	1120.39±157.14^b^	0.543±0.07^b^	165.98±12.47^b^
Lovastatin	1354.39±124.17^a^,^b^	0.587±0.06^a^,^b^	110.35±11.27^a^,^b^
Zhibituo	1368.27±124.77^a^,^b^	0.588±0.08^a^,^b^	78.98±8.74^a^,^b^
Low-dose XMJ	1452.33±102.47^a^,^b^	0.622±0.07^a^,^b^	79.54±6.57^a^,^b^
Middle-dose XMJ	1688.69±149.38^a^,^b^	0.636±0.08^a^,^b^	63.21±4.57^a^,^b^
High-dose XMJ	1657.69±123.96^a^,^b^	0.654±0.07^a^,^b^	62.17±6.46^a^,^b^

Data shown are the mean ± standard error.

a P<0.05 vs. model group;

b P<0.05 vs. blank control group.

TIMP-2, tissue inhibitor of metalloproteinase-2; MMP-2, matrix metalloproteinase-2; NF-κB, nuclear factor, κB; XMJ, Xin Mai Jia.
